# On Amalgams

**Published:** 1874-10

**Authors:** Amos Kirby


### ARTICLE V.

### *On Amalgams.*

BY AMOS KIRBY, ESQ., L. D. S.

In fulfilment of my promise, I send a short account of some experiments with amalgams for stopping teeth, and which I have not published except as a casual communication to the Odontological Society.

Before Mr. C. S. Tomes' interesting experiments on the changes in specific gravity in amalgams during hardening, I believe no attempt had been made to determine with exactness the behaviour of those substances.

Wishing to verify Mr. Tomes' results by other methods, as well as to make further practical investigations, I had apparatus made of two or three kinds for measuring very small changes of bulk. The most useful of these was a small screw micrometer attached to the movable end of a metal box, which could be filled with samples of the substance to be observed, so that any changes in their diameter could be measured, and as the pieces could be readily removed and replaced again, they could be re-measured at any interval of time.

Some of the stoppings in general use were tried, and found to contract, as a rule, but not always to the same extent; one of the best did so usually to about 16–10,000th (.0016) of its diameter; but in these and all other amalgams tried, whether they expanded or contracted, the changes went on for some hours, or even days, after they had become perfectly hard.

Precipitated silver amalgam being the only substance of the kind which had been supposed to expand, was teste l, and some samples did so to the extent of about 1–40th of their diameter, (.025,) but contrary to expectation, pure filed silver also acted in exactly the same way, expanding for several days. From this I supposed that amalgams made with more silver (in proportion to the tin) than has been ordinarily used, would probably expand; and found that such was the case. A large excess of silver, however, made the amalgam too brittle, and caused discoloration, whilst better results followed with a small excess of that metal.

The best results seemed to be obtained when the quantities of silver and tin bore relation to their atomic weights, and the addition of a little gold made the paste more plastic as well as causing it to set more rapidly.

Two atoms of silver to one of tin, with about seven per cent. of gold, produced, with an equal weight of mercury, (more being required if the fillings are new,) an amalgam which expanded from 16–10,000ths to 50–10,000ths, an amount sufficient to insure (other things being favorable) perfect tightness in a plug without causing danger to a frail tooth.

Amalgam made from an alloy in the proportion of three atoms of silver to two of tin, with seven per cent. of gold, expanded slightly, and seems to stand well in the mouth without discoloring much or being too brittle—say,

| | |
|---|---|
| Silver - - - - - - | 162 grains. |
| Tin - - - - - - - | 118 " |
| Gold - - - - - - | 20 " |

Or, roughly—

| | |
|---|---|
| Silver - - - - - - - | 4 dwts. |
| Tin - - - - - - - | 3 " |
| Gold - - - - - . - | $\frac{1}{2}$ " |

These are melted and reduced to fillings, which should not be used for two or three weeks; about an equal weight of mercury is added to form a paste.

This and other amalgams containing an excess of silver expand, as a rule, but not always to the same extent—and sometimes even contract slightly, or contract first and expand afterwards, or they occasionally expand first, or contract first, and then regain and retain their original size.

An unexpected phenomenon of a very important nature followed the hardening of the first samples of stopping tested in the micrometer box and in all, which were used in a dry condition, but was particularly remarked in some new kinds which were said by the vendor not to change in shape at all when used in a dry state; i. e. with very little mercury. It consisted in so great a change in the shape of the mass or bar of stopping after hardening, as often to prevent its being again introduced into the box for measurement, and was always attended by a contraction and shrinking of the middle part of the surface, (the portion last introduced)

with a turning up and in toward the centre of the edge of the mass. When the mass was in the form of a long bar, the ends bent up like a bow.

This tendency to change of shape proved to be independent of the composition of the stopping, and of its general expansion or contraction, and depended entirely on the quantity of mercury with which it was mixed, or rather on the unequal distribution of the mercury through the mass when only a small proportion of it was used. It is in itself fatal to the efficiency of any stopping in which it takes place, but may be prevented or reduced to a minimum by care in packing, using a moderately soft paste, and drying the mercury out of the surface of the stopping with a very dry lump of amalgam used as a sponge, squeezing out the mercury it has absorbed, and applying it again and again.

It appears that when the amalgam is used in a dry state, the pressure of the instrument in packing, squeezes some of the mercury to the surface, however little there may be in it, so that the last portion of stopping introduced contains more of that substance than the first; some time afterward, however a more equal distribution takes place—the first or dryer portion taking away mercury from the latter portion, expands in the process, whilst the part which has lost mercury contracts in consequence.

If, however, the first part is soft when introduced, it is easy to use the last portion dryer, and so insure equal distribution. In practice I found that the dryer the amalgam the greater the distortion ; and that when used as stated above, little or no change followed.

Another test which I contrived consisted, in its first form, in introducing amalgam into short pieces of glass tube closed at one end, in order to see if any air space was formed between the two ; some of the shrinking amalgams fell out of these tubes after a few days, but those made of pure silver occasionally split the tubes in two, although in some cases the tube remained unbroken, while the silver projected from the open end, and after being ground off level, projected again next day.

These tubes were afterwards placed in a vessel containing Judson's blue dye, but unless the shrinkage in the amalgam had been very great, the color did not find its way between the glass and the stopping; the vessel containing them was therefore put under an air pump, and after partial exhaustion of the air, the color found its way into all those which were not perfect. Some tubes filled with silver amalgam were quite proof against the color, and some with the expanding amalgam named above were good when the surface had been well dried from mercury, but none of the bought fillings stood the test, and great care was needed to enable any to do so.

From this experiment it is evident that the "ink test," as recommended by Mr. Fletcher, is not reliable, and I am sorry to find by the micrometer test, that a sample of his new "expanding amalgam," which has no doubt been tested by it, is by no means perfect, but contracts when used with a moderate quantity of mercury.

Amalgams with a large quantity of silver have long been used with the same method of drying the surface, and in many cases useful results have followed the less careful use of imperfect materials, but it is certain that all amalgams must be looked upon as capricious in their behavior and not thoroughly reliable.

As we are yet without a reliable plastic filling, the newest and best of the oxychlorides dissolving in a year in some mouths, whilst it endures much longer in others, the relative merits of gold used in its different forms is a question of the the greatest importance, and it is satisfactory to know that experiments are being made by able hands with a view to deciding the subject. Judging from some of the physical properties of the two, I have long thought that the use of non-adhesive gold is to be preferred, but chiefly from the ease with which it may be condensed (a process which in this case may be compared to the caulking of a wooden ship,) by which moisture is perfectly excluded if the gold has been previously made fairly solid. Although the adhesive stopping may be much harder and more solid, it is like the hard material of an iron ship or steam boiler, the seams of which appear to be quite close after they are riveted, whilst in fact, they readily allow the passage of moisture. In the iron ship this is remedied by a process of caulking different to the one used for wooden vessels, and inapplicable in the case of teeth stopped with adhesive gold, from that substance being harder than the tooth, which, from its brittleness, is unable to bear the force necesssary for efficient caulking of the harder substance.

There are not many tooth cavities of a form which cannot be filled with soft gold, and since pellets of from one to two grains of No. 6 foil may be packed or made solid by the aid of the mallet, before introducing more, it is not difficult to build large stoppings rapidily, and when the surface has been well hammered it is a matter of some labor to cut it away for the accommodation of the cusps of an antagonizing tooth.

Without being able to throw any new light on the processes used in making non-adhesive stoppings, it is well to call them to mind for use in some cases where one might without them be tempted to use amalgam. The mallet, both for building and condensing large fillings cannot be too highly prized, and for the former process the straight lever mallet instrument, of which I some time ago gave patterns to Messrs. Ash and Collins, answers every purpose; while for condensing the surface of crown cavities, I have used special instruments of simple construction with very short blades; these are very much more efficient than any of those in ordinary use, which from the curves in the shaft necessary to enable the operator to reach the part, are quite inefficient, anything beyond the slightest bend acting as a spring and absorbing all the force of the blow. The mallets named have not been made except for my own use. They are very convenient, as they enable the operator to condense from behind forward, and I shall be happy to give drawings of them.—*British Jour. of Dental Science.*

